# Barriers to Safe Oxygen Therapy and the Effect of the Training on the Knowledge and Performance of ICU Nurses

**DOI:** 10.1155/2023/5490322

**Published:** 2023-11-09

**Authors:** Samaneh Mirzaei, Mohsen Gholinataj Jelodar, Shahab Rafieian, Farzaneh Sadat Dehghan, Asma Jaafari Nia, Khadijeh Nasiriani, Amir Neshati

**Affiliations:** ^1^Clinical Research Development Center, Shahid Rahnemoun Hospital, School of Medicine, Shahid Sadoughi University of Medical Sciences, Yazd, Iran; ^2^Department of Health in Emergencies and Disasters, School of Public Health, Shahid Sadoughi University of Medical Sciences, Yazd, Iran; ^3^Department of Internal Medicine, School of Medicine, Shahid Sadoughi University of Medical Sciences, Yazd, Iran; ^4^Department of Thoracic Surgery, Imam Khomeini Hospital, Tehran University of Medical Sciences, Tehran, Iran; ^5^Nursing Department, Nursing and Midwifery Research Center, Maternal and Newborn Health Research Center, Shahid Sadoughi University of Medical Sciences, Yazd, Iran; ^6^Industrial Diseases Research Center, Center of Excellence for Occupational Medicine, Shahid Sadoughi University of Medical Sciences, Yazd, Iran

## Abstract

**Introduction:**

Proper oxygen therapy is crucial in hospitals, particularly intensive care units, to ensure safety and accuracy. The role of nurses during oxygen therapy is vital, as their knowledge and correct performance significantly impact patients' clinical conditions. A study was carried out to examine the knowledge and performance of nurses regarding safe oxygen therapy. The study aimed to identify the obstacles hindering safe oxygen therapy and assess the impact of training on the knowledge and performance of intensive care nurses.

**Methods:**

This study was conducted among the ICU nurses at Shahid Rahnemoun Teaching Hospital in Yazd, Iran. The study method is a sequential combination of descriptive, qualitative, and educational phases. The first stage involved examining the knowledge and performance of 80 ICU nurses in oxygen therapy. The study employed content analysis to elaborate on participants' perspectives on safe oxygen therapy challenges and potential solutions. The third phase involved a two-group study with pre- and post-tests to examine the effect of training on ICU nurses' knowledge and performance in oxygen therapy.

**Results:**

The study found that intervention and control groups had low average scores in knowledge, performance, and total score of oxygen therapy before the study, with no significant difference. There was a significant difference between intervention and control groups one and three months after the intervention in the areas of knowledge (after-1 month 24.41 vs. 20.29, 95% CI [3.144–5.098], after-3 month 22.13 vs. 20.24, 95% CI [0.729–3.053]), performance (after-1 month 21.54 vs. 18.05, 95% CI [2.898–4.073], after-3 month 19.74 vs. 18.63, 95% CI [0.400–1.824]), and total score of oxygen therapy (after-1 month 45.95 vs. 38.34, 95% CI [6.288–8.925], after-3 month 41.87 vs. 38.87, 95% CI [1.394–4.613]).

**Conclusion:**

The study's findings revealed that nurses in ICUs lack the appropriate knowledge and performance in oxygen therapy. A lack of knowledge and correct practice, insufficient monitoring of oxygen therapy, and defects in hospital equipment are contributing factors. The training was found to improve the knowledge and performance of nurses significantly. Consistent training at shorter intervals is suggested for nurses to keep their knowledge current.

## 1. Introduction

Oxygen therapy for various diseases started at the end of the 18th century and is used for many medical treatments today [1]. Oxygen is a drug prescribed to patients with different health conditions, with doses and concentrations adjusted based on clinical requirements [2, 3]. The administration of oxygen relies on the patient's needs and the medical team's assessments [2]. Various devices are available for oxygen therapy in hospitals [4–6]. Nurses should follow the same protocol with this treatment as with other drug therapies and only administer it with a printed and direct physician order [7]. The doctor's prescription must specify the oxygen therapy equipment, treatment procedure, patient dosage, and target oxygen saturation level [1, 8].

It should be noted that oxygen therapy is beneficial when used with the correct dose and method [2]. Like any other medication, the inappropriate use of this drug can lead to adverse effects, such as hypotension, atelectasis, retrolental fibroplasia, and nasal mucosa irritation [1, 9, 10].

Lung tissue is affected by oxygen. High oxygen concentrations inhibit surfactant production, leading to the alveoli's collapse and reduced gas exchange on the alveoli-capillary surface [11, 12]. Inappropriate oxygen prescriptions can result in more extended hospital stays, higher ICU admission rates, and an increased risk of death [7]. Therefore, patients should receive this treatment appropriately, safely, and conveniently [2]. To attain this objective, staff should acquire the essential expertise to determine the appropriate oxygen delivery method based on the patient's requirements and with a secure therapeutic quantity [3]. Thus, they should continuously monitor oxygen administration and gradually reduce supplemental oxygen to eliminate these risks [11].

Regular monitoring of patients is essential during oxygen therapy, and nurses play a vital role in it [13]. They need to be familiar with the basic principles, indications, methods of use, complications of oxygen therapy, and the causes of hypoxemia. Patients receiving oxygen therapy require proper care, and their care measures should be recorded [1, 10].

Like other drugs, nurses may face barriers when administering oxygen to patients. Barriers to proper oxygen therapy may be attributed to nurses' inadequate knowledge of devices and hospital-related obstacles, such as lack of training or access to equipment and protocols [14].

A couple of studies evaluated nurses' knowledge of oxygen use and found their scores to be low [15, 16]. Issues related to inadequate knowledge, attitude, and practice regarding oxygen administration still need to be addressed for adequate oxygen therapy. Nurses' skills are crucial to delivering quality care [11]. Many educational interventions in nursing are based on the assumption that knowledge acquisition improves clinical decision-making. Identifying nurses' knowledge level about oxygen therapy helps to determine the need for training and improve performance [17].

This study examined the knowledge and performance of nurses in the ICU regarding oxygen therapy, as well as the barriers and solutions associated with its safe administration.

## 2. Methods

This study was conducted among the Shahid Rahnemoun teaching hospital ICU nurses in Yazd, Iran. This hospital has four ICUs and thirty-six beds for trauma and nontraumatic medical patients. The study method involves a sequential combination of descriptive, qualitative, and educational phases. The first phase involved assessing the knowledge and performance of ICU nurses in oxygen therapy through a questionnaire. The second phase used the qualitative method to explore the views of nurses and specialists on the barriers to safe oxygen therapy in hospitals and the proposed solutions to achieve this goal. Next, in the third phase, a two-group quasi-experimental design with pre- and post-tests was used to explore the level of knowledge and performance in oxygen therapy among ICU nurses.

**Figure 1:**
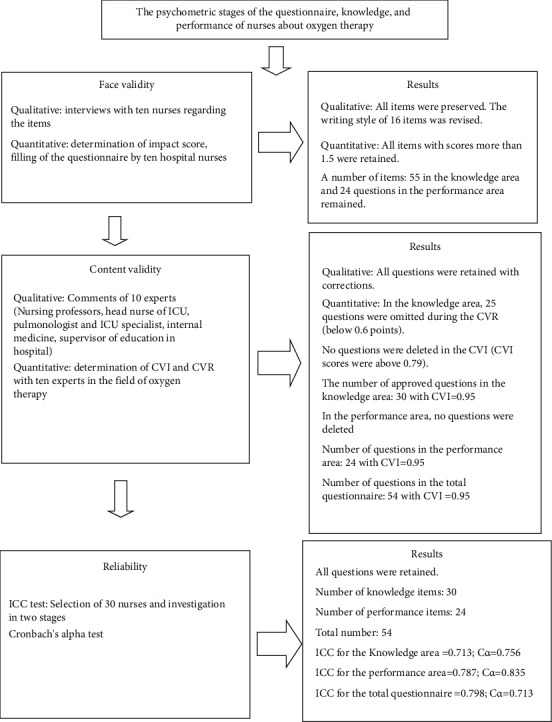
Psychometric questionnaire knowledge and performance of nurses in oxygen therapy.

The tool is design to designed and determine its validity and reliability. Nurses' knowledge and performance were evaluated by extracting items from nursing textbooks and articles related to oxygen therapy [1, 2, 17–21]. At first, 101 items were pulled by the research team; after removing duplicate items, 55 items were confirmed in the area of knowledge. In the performance section, the items mentioned in the principles and techniques of nursing in the field of oxygen therapy and the articles [1, 2, 17–21] were used. 30 items were extracted by the research team (including the nursing professor, head nurse of the ICU, pulmonologist, ICU specialist, and supervisor of education in the hospital) for the performance section. After removing duplicates, the research group confirmed and applied 24 items after discussing and reviewing the performance area. Then, the validity and reliability of the questionnaire were examined. The validity and reliability of the questionnaire were established through face validity (quantitative and qualitative) and content validity (quantitative and qualitative), as well as internal consistency and stability testing. For internal consistency, Cronbach's alpha index (C*α*) was used to calculate the correlation between the questions in each dimension and the entire questionnaire. The ICC (intraclass correlation index) [22] was utilized to evaluate the tool's stability over time (test-retest), with a 95.0% confidence interval. For this purpose, the tool designed by a sample of 30 nurses in two stages was completed with a time interval of two weeks. Usually, alpha values higher than 0.7 are considered to be a sign of the acceptable reliability of the tool [23]. After verifying its validity and reliability, the questionnaire was designed to measure nurses' knowledge, performance, and demographic information. The knowledge section and the checklist of the performance section related to the oxygen therapy of nurses were approved (Figure 1).

### 2.1. Descriptive Phase

At this phase, nurses in the study were selected based on the available sample size. The sample size was determined using Cochran's formula, resulting in 73 people from a statistical population of 90 nurses across 4 ICUs. In anticipation of a sample loss of 10%, the sample size was increased to 80 individuals. The study included ICU nurses with B.S./MSc nursing degrees and at least six months of work experience. Those who worked part-time, refused to participate, or were transferred to other departments were omitted. The nurses' knowledge and performance were assessed through a questionnaire. The nurses responded to questions about their knowledge while the researcher checked off the performance section. It was checked by the same nurse on the morning shift of nurses.

The data collection tool has three sections: demographic information, knowledge, and performance area. The demographic information encompasses age, gender, marital status, ICU work experience, type of ICU, educational level, and completion of an ICU course. The knowledge section comprises 30 questions covering indications of oxygen therapy, oxygen poisoning, different devices for oxygen therapy, and precautions. One point will be awarded for each correct answer, while incorrect ones will receive no score. The range of the field of knowledge's score is from 0 to 30. The performance checklist comprises 24 items. One point will be awarded if it is executed correctly. The performance is rated on a scale of 0–24. The questionnaire has a total score of 0–54 points. The research team determined the validity and reliability of the questionnaire with 0.95 and 0.71, respectively.

Nurses' knowledge and performance were evaluated by extracting items from nursing textbooks and articles related to oxygen therapy [1, 2, 17–21]. At first, 101 items were pulled by the research team; after removing duplicate items, 55 items were confirmed in the area of knowledge. In the performance section, the items mentioned in the principles and techniques of nursing in the field of oxygen therapy and the articles [1, 2, 17–21] were used. 30 items were extracted by the research team for the performance section. After removing duplicates, the research group confirmed and applied 24 items after discussing and reviewing the performance area. Then, the validity and reliability of the questionnaire were examined. The validity and reliability of the questionnaire were established through face validity (quantitative and qualitative) and content validity (quantitative and qualitative), as well as internal consistency and stability testing. For internal consistency, Cronbach's alpha index (C*α*) was used to calculate the correlation between the questions in each dimension and the entire questionnaire. The ICC (intraclass correlation index) [22] was utilized to evaluate the tool's stability over time (test-retest), with a 95.0% confidence interval. For this purpose, the tool designed by a sample of 30 nurses in two stages was completed with a time interval of two weeks. Usually, alpha values higher than 0.7 are considered to be a sign of the acceptable reliability of the tool [23]. After verifying its validity and reliability, the questionnaire was designed to measure nurses' knowledge, performance, and demographic information. The knowledge section and the checklist of the performance section related to the oxygen therapy of nurses were approved (Figure 1).

### 2.2. Qualitative Phase

Based on the previous step's results of nurses' knowledge and performance, the views of nurses and experts on barriers to safe oxygen therapy and recommendations to achieve it were presented. Conventional content analysis was utilized in this qualitative study through semistructured interviews held from January 2023 to February 2023.

The primary data collection method in this research was semistructured interviews. To conduct the study, the researcher established a proper relationship, informed the participants about the interview's duration, obtained informed consent, and recorded the conversations. Confidentiality of information and interview files was assured to the participants during the study. The interviewee requested further explanation or confirmation if there was any ambiguity during the conversation. The questions were designed to fit the research's purpose and serve as an interview guide. The order of questions changed per the participant's responses and the study's stages. The interview began with general questions and then proceeded based on the participants' responses. The questions for the interview were selected based on an analysis of previous discussions. The process of sampling continued until data saturation.

Questions such as “What are the barriers to safe oxygen therapy from your point of view?” “In your opinion, what solutions implement safe oxygen therapy?” were asked. The interview was able to progress smoothly due to these open-ended questions. The qualitative data were analyzed using Granheim and Lundman's conventional content analysis method (2004) [24]. All contents (notes and recorded audio files) were transcribed word for word at the end of each interview. The typed interviews were reviewed multiple times and broken down into minor semantic units or codes. The primary codes were compared and grouped into subcategories based on their similarities.

The main category was extracted by continuously comparing subthemes according to their compatibility and similarity. The interviewer provided feedback to ensure the accuracy of the interviewee's impressions and prompted them to elaborate and give examples as needed. In this research, the Lincoln and Guba criterion [25] was used to increase the accuracy and strength of the data. To ensure data credibility, the researcher established a long-term relationship with participants and aimed for maximum diversity in knowledge, experience, work length, service places, age, and gender. The data's reliability was boosted by the step-by-step repetition of data collection and analysis and the consultation of the research team and experts. Also, a constant analysis was conducted, and the data were collected, coded, and analyzed simultaneously. A summary of the interview was given to the interviewees after the implementation to verify its accuracy. The Prolong Engagement method was utilized to ensure validity and acceptability.

In addition, the member check technique was utilized to compare the researcher's interpretation with the participants' intended meaning, and the interview summary was given back to the participants to ensure the interview's precision. Qualitative experts and the author evaluated the codes throughout the coding process. The duration of the interviews was in the range of 20 to 60 minutes. The interviewees chose the location for the interviews, mostly at their workplaces. Experts with differing viewpoints spent most of their time extracting codes. Numerous meetings were held to establish a rational connection between codes and categories.

### 2.3. Educational Phase

Based on the qualitative phase of the study, one of the barriers to safe oxygen therapy was the need for more training. On the other hand, according to the results of the descriptive phase of the study, the majority of nurses were at an unfavorable level of knowledge and performance in the field of oxygen therapy, so at this stage, the effect of training interventions on the knowledge and performance of ICU nurses was investigated. At this study stage, nurses who participated in the research's descriptive phase were considered for inclusion criteria. Participants who failed to attend all training sessions or complete the questionnaire were excluded. Eighty nurses were randomly assigned into intervention and control groups using allocation software during the descriptive phase.

The educational supervisor and department officials in the intervention group made arrangements for shift planning, enabling nurses to attend the classes. The classes were planned with necessary arrangements such as location, schedule, educational materials, and reception. The educational content was presented by the researchers in the form of lectures, discussions, questions and answers, and demonstrations. Over two consecutive days, a 4-hour workshop provided educational content on respiratory system anatomy and physiology, indications for oxygen administration, oxygen poisoning, and nursing care for patients receiving oxygen, complications of incorrect oxygen therapy, familiarity with oxygen therapy devices, and their proper use. The control group did not receive any training. The descriptive phase scores were utilized to determine the nurses' knowledge and performance scores before the training intervention. The intervention and control groups of nurses were assessed for knowledge and performance levels one and three months after the training sessions. Upon completing the study, the educational package was given to the control group for reading.

The ethics committee authorized the study at Shahid Rahnemoun Hospital, Yazd, with the ethics I.D. I.R.SSU.SRH.REC.1400.010. The nurses involved in the study provided written informed consent after being informed of the study's objectives and the confidentiality of the information.

Data were analyzed using SPSS version 26 software. Descriptive statistics included absolute and relative frequency, mean, and standard deviation. The inferential statistics used included an independent *t*-test, an ANOVA with repeated measures, and a chi-square with a 95% confidence interval.

## 3. Results

The study's findings were shown in three phases: descriptive, qualitative, and educational.

### 3.1. Descriptive Phase

At this phase, data were analyzed on 80 nurses participating in the study. The average age of the participants in this phase was 32.75 ± 6.14 years, with a work experience of 8.83 ± 4.67 years. The majority of the study groups were female (76.3%) and single (56.3%) and had a bachelor's degree (87.5%).  Table 1 displays the average knowledge, performance, and total score of oxygen therapy in the units studied. Based on percentile agreement, scores above 25% were deemed favorable, scores in the bottom 25% were unfavorable, and scores in the middle 50% were average.

Table 2 shows the degree of nurses' awareness about the person responsible for starting, determining the dose, type of oxygen therapy device, and oxygen consumption cessation.

### 3.2. Qualitative Phase

A total of 13 participants were selected purpose-based for the qualitative phase of the study, including 2 emergency medicine specialists, 3 pulmonary and ICU specialists, four hospital supervisors and ICU head nurses, and four experienced nurses with more than ten years of work experience. Maximum variation sampling continued until data saturation. The average age of the participants in the qualitative phase was 39.23 ± 2.20. Based on the content analysis, four main categories include lack of knowledge in the field of oxygen therapy (with six subcategories), lack of sufficient monitoring of the oxygen therapy process (with five subcategories), defects in the correct implementation of oxygen therapy at the patient's bedside (with five subfloors), quantitative and qualitative defects of oxygen therapy devices (with four subcategories) were extracted as barriers to safe oxygen therapy (Table 3).

**Figure 2:**
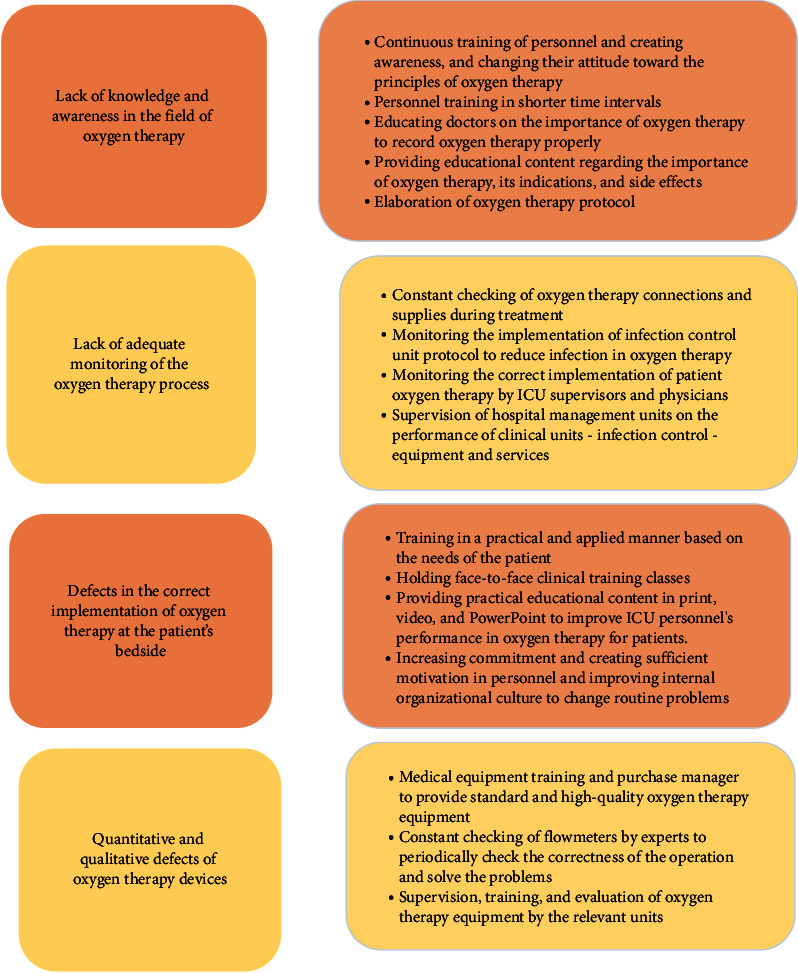
Appropriate recommendations to barriers to safe oxygen therapy.

Also, the appropriate recommendations to solve these barriers were explained based on the interviews conducted (Figure 2).

### 3.3. Educational Phase

In this phase, 80 nurses in the descriptive phase were divided into two groups (40 people in each intervention and control group). During this stage, one participant from the intervention group and two from the control group were removed from the study due to missed training sessions and incomplete questionnaires. The data was analyzed on 39 nurses in the intervention group and 38 in the control group.

Demographic information is presented in Table 4. The chi-square and independent *t*-test results indicated no significant differences between the two groups regarding age, sex, work experience in ICU, work experience in COVID ICU, level of education, and completion of the oxygen therapy course.

The interventional group showed significant differences in all areas when comparing the average scores of knowledge and performance and the total score of oxygen therapy three times (before training, one month, and three months after intervention). However, the scores in three-time points showed no significant difference in the control group. Results are shown in Table 5. The two-by-two comparison of measurement time points in the intervention group demonstrated a significant difference between the three times in knowledge and performance scores, as indicated by the Bonferroni post hoc test. The control group did not report any significant differences.

The study's results show no statistically significant differences between the intervention and control groups' knowledge, performance, and total scores on oxygen therapy before the study. It was reported to be significant after the survey (one month and three months later). The results are shown in Table 6.

## 4. Discussion

Our study revealed that ICU nurses need a better understanding and performance in oxygen therapy, with only 12.5% having adequate knowledge and 25% displaying favorable performance. Proper understanding and implementation of oxygen therapy were observed in only 11.25%. Most ICU nurses need more knowledge and performance status for oxygen therapy, which is unacceptable given its importance in these departments. These findings align with other studies in the knowledge and performance of nurses [1, 17, 19, 20, 26–28].

Nurses should only administer oxygen therapy with a doctor's order, specifying the device, process, dose, and target oxygen saturation level [1, 7, 8]. For deciding on a prescription, dose, device, and ending oxygen use, the percentages of nurses who were aware of the responsible person were 38.8%, 40%, 47.5%, and 52.2%, respectively. These findings reveal that many nurses need more awareness in this domain.

The study found that the primary barriers to safe oxygen therapy are insufficient knowledge and performance, inadequate monitoring, and defects in oxygen therapy equipment. The present study is in agreement with previous studies [2, 3, 13, 20, 26] that identified nurses and healthcare care professional's insufficient knowledge about oxygen therapy, absence of oxygen therapy protocol, and lack of oxygen therapy equipment as the primary challenges in ensuring safe oxygen therapy. The study recommends continuous, shorter training to raise awareness and change attitudes towards oxygen therapy principles, properly monitoring oxygen therapy, fixing equipment defects, and motivating nurses to improve their performance. The results of this research, along with other studies [3, 29, 30], highlight the necessity for more education for nurses on oxygen therapy and the knowledge deficit among nurses, physicians, and other healthcare professionals regarding O2 administration.

This study aimed to enhance the knowledge and practice of oxygen therapy in ICU nurses through training. Following the intervention, the group of nurses showed a substantial improvement in their knowledge and performance in oxygen therapy, which persisted in both the short term (one month) and long term (3 months). Oxygen therapy training positively affected the knowledge and performance of nurses, as evidenced by a statistically significant difference, which is in line with other studies [17, 19]. The comparison between one month and three months revealed that although there was an improvement in knowledge and performance after the intervention, a noticeable gap was still observed in the third month in contrast to the first month. Nurses' knowledge and performance decreased after three months compared to the first month. Education is crucial to enhancing the knowledge and performance of nurses, but for its long-term sustainability, nurses need more frequent and concise training sessions. It is shown that the oxygen therapy training program has improved nurses' clinical competence in oxygen therapy. To attain the highest score, executing a continuous training program is crucial. Educational programs should be repeated to stay effective and updated with the latest changes in the clinical manual of oxygen therapy. Undergraduate nursing students and special nursing master's degrees should learn the clinical manual of oxygen therapy. Nursing managers should improve nursing services per the oxygen therapy clinical guide to ensure safe and proper oxygen therapy. It is proposed that a trained resident physician should be present to instruct ICU nurses. If not available, it is crucial to establish a particular protocol for oxygen therapy to avoid errors.

### 4.1. Limitations

The study faced limitations with adjusting the education plan and nurse schedules, but attempts were made to schedule classes on less busy working days. Contact between nurses from the interventional and control groups was outside the researcher's control.

## 5. Conclusion

ICU nurses need more favorable knowledge and optimal performance in oxygen therapy, and this is compounded by insufficient monitoring of the oxygen therapy process and quantitative and qualitative defects of oxygen therapy devices, which pose barriers to safe oxygen therapy. Knowing these components and working to eliminate any barriers is important to promote safe oxygen therapy for patients. The impact of training was highlighted as a vital aspect in ameliorating nurses' understanding and proficiency and safeguarding their standing in this domain.

## Figures and Tables

**Table 1:** Comparison of mean and standard deviation of knowledge, performance, and total score of oxygen therapy in the studied units.

Area	Total M ± SD	Level	*N* (%)	Min-max score	M ± SD by levels
Knowledge (0–30)	20.29 ± 2.41	Unfavorable (19≥)	30 (37.5)	13–19	17.90 ± 1.60
		Medium (20–22)	40 (50)	20–22	21.10 ± 0.77
		Favorable (>22)	10 (12.5)	23–26	24.20 ± 1.22

Performance (0–24)	18.26 ± 1.56	Unfavorable (17≥)	28 (35)	15–17	16.54 ± 0.69
		Medium (18–19.75)	32 (40)	18-19	18.50 ± 0.50
		Favorable (19.76–24)	20 (25)	20–23	20.30 ± 0.73

Total score (0–54)	38.55 ± 3.75	Unfavorable (36≥)	25 (31.25)	30–36	34.28 ± 1.83
		Medium (37–42)	46 (57.5)	37–42	39.70 ± 1.84
		Favorable > 42	9 (11.25)	43–49	44.56 ± 2.12

M: mean; SD: standard deviation; *N*: number.

**Table 2:** The level of awareness of the decision-maker for oxygen therapy in the studied units.

A decision maker for oxygen therapy	Physician		Nurse		Both	
	*N*	%	*N*	%	*N*	%
Prescribing oxygen therapy	31	38.8	8	10	41	51.2
Determining the dose of oxygen therapy	32	40	16	20	32	40
Choosing an oxygen therapy device	38	47.5	6	7.5	36	45
Stop oxygen therapy	42	52.5	14	17.5	24	30

**Table 3:** Extraction of main categories and subcategories of barriers to safe oxygen therapy from the point of view of the study units in the qualitative phase.

Barriers to safe oxygen therapy		Expressions of the interviewees
Categories	Subcategories	
Lack of knowledge and awareness in the field of oxygen therapy	(i) Lack of sufficient knowledge and awareness of the personnel about the importance of correct and safe oxygen therapy and its side effects	…“The education that is given does not say everything that is needed in nursing work...” (p. 12)
	(ii) Lack of continuous training in terms of quantity and quality	...“Clinical training is not given; nurses mostly do not know which equipment to use for which patient...”(p. 10)
	(iii) Absence of oxygen therapy training protocol in the hospital	…“They don't know if they should pour distilled water or wash these oxygen containers at all...” (p. 1)
	(iv) Lack of knowledge of some physicians about the importance of correct and safe oxygen therapy	...“ Some nurses do not know at all what this patient's clinical condition is and how much oxygen he should receive…” (p. 13)
	(v) Lack of knowledge of the personnel about the indications of oxygen therapy prescription, dose, target, and duration of its use	…“When, as a nurse, I ask some physicians about the dose and equipment of oxygen therapy, they call me ignorant and say that I have no expertise in this field and just coordinate with the ICU manager...”(p. 3)
	(vi) Lack of knowledge of personnel and services staff about the hygiene principles of infection control in oxygen therapy	
	(vii) Lack of knowledge of patient examination methods to assess oxygen saturation in the blood	

Defects in the correct implementation of oxygen therapy at the patient's bedside	(i) Failure to include the oxygen therapy ordered by the attending physician in full with the type of device and required dose in the patients' files	…“Sometimes I see nurses tape the venturi mask adapter so that more oxygen reaches the patient...” (p. 2)
	(ii) Unnecessary oxygen administration without a physician's order	...“When you come to visit, you see that oxygen is given unnecessarily to a patient who does not need oxygen without an order...” (p. 7)
	(iii) Lack of coordination between services and nurses in the field of oxygen therapy implementation	...“Sometimes the service staff, when they change the sheets or move the patient, the mask falls off the patient's face, and they forget to put it on...” (p. 4)
	(iv) Failure to use oxygen therapy equipment correctly (type of equipment, dosage, length of connections)	...“These containers that should be filled with distilled water or they don't wash the container at all...” (p. 6)
	(v) Failure to properly implement the protocol of the infection control unit	...“When you look at the patient with a reservoir mask, they give oxygen, but the oxygen flow is 2 liters...” (p. 13)
		…“Physicians either give oral orders for oxygen or do not write the dosage and the device at all...” (p. 1)

Quantitative and qualitative defects of oxygen therapy devices	(i) Inconsistency and lack of attention in purchasing medical equipment	“This flowmeter gives so little oxygen that you have to take the flow to 10 liters for the nasal tube to give a little oxygen...” (p. 12)
	(ii) Errors in the correct operation of flowmeters	“Sometimes I know that I shouldn't reserve masks now, but we don't have simple masks in the department, and when I call the pharmacy, they say that there are too many masks and we won't buy masks until they run out...” (p. 5)
	(iii) Leakage or malfunction of the flowmeter adjustment column and the ball inside the flowmeter	“These oxygen pressure alarms sounded so much sometimes, especially during the time of corona, that we had to lower the oxygen flow for the patients...” (p. 3)
	(iv) Lack of proper oxygen generator	“This ball in the flowmeter doesn't go up and down at all in some flowmeters, and I don't understand how much the flow is now...” (p. 6)

Lack of adequate monitoring of the oxygen therapy process	(i) Lack of head nurse supervision	…“Sometimes, when the services fill the oxygen distilled water, they raise or lower the oxygen flow themselves, and the nurses don't pay attention at all...” (p. 5)
	(ii) Lack of adequate monitoring of oxygen therapy by the patient's nurse	“...ICU resident doctors or supervisors do not look at all to see if the patient has an oxygen order or not or if this device is suitable for this patient...” (p. 11)
	(iii) Lack of supervision by the resident doctor in the ICU	...“The infection control unit should come and collect culture samples from these containers that pour distilled water, but it cannot be done at all...” (p. 9)
	(iv) Inadequate supervision of the infection control unit on compliance and implementation of the hygiene principles of infection control in oxygen therapy	... “It seems that no one confirms the quality of the equipment they buy...” (p. 8)
	(v) Lack of intraorganizational supervision at management levels on all units (clinic-medical equipment-infection control unit)	...“There is no supervision by the people in charge of the hospital on the quality and quantity of the purchase of equipment or the work of the service department...” (p. 4)

P: participant.

**Table 4:** Demographic characteristics of the studied units in the educational phase.

Variables		Intervention group	Control group	*P* value
Age, year	Mean ± SD	33.33 ± 7.20	31.4 ± 4.90	0.319
Work experience, year		9.33 ± 4.93	8.24 ± 4.57	0.316
Work experience in COVID ICU, month		6.72 ± 8.68	6.84 ± 8.76	0.950

Sex	*N* (%)			
Male		6 (15.38)	11 (28.95)	0.151
Female		33 (84.62)	27 (71.05)	
Marital status				
Single		23 (58.98)	20 (52.63)	0.575
Married		16 (41.02)	18 (47.37)	
Level of education				
Bachelor of science		33 (84.62)	34 (89.47	0.526
Master of Science		6 (15.38)	4 (10.52)	
Passing the course of oxygen therapy				
Yes		11 (28.20)	6 (15.78)	0.189
No		28 (71.80)	32 (84.22)	
Work experience in the COVID ICU				
Yes		29 (74.36)	28 (73.68)	0.947
No		10 (25.64)	10 (26.32)	

*N*: number; SD: standard deviation; %: percent.

**Table 5:** Comparison of the mean scores of knowledge and performance and total score of oxygen therapy between two groups at different times of the study.

Groups	Variables	Before mean ± SD	After-1 month mean ± SD	After-3 months mean ± SD	*F*	*P* value^*∗*^
Intervention group	Knowledge (0–30)	20.10 ± 2.59	24.41 ± 2.06	22.13 ± 2.76	29.19	*P* < 0.0001
	Practice (0–24)	18.03 ± 1.67	21.54 ± 1.46	19.74 ± 1.51	49.68	*P* < 0.0001
	Total (0–54)	38.13 ± 3.98	45.95 ± 2.87	41.87 ± 3.76	46.69	*P* < 0.0001

Control group	Knowledge (0–30)	20.47 ± 2.34	20.29 ± 2.24	20.24 ± 2.33	0.111	*P* = 0.895
	Practice (0–24)	18.58 ± 1.50	18.05 ± 1.08	18.63 ± 1.61	1.93	*P* = 0.15
	Total (0–54)	39.05 ± 3.62	38.34 ± 2.93	38.87 ± 3.30	0.623	*P* = 0.623

*P* value^*∗*^ = ANOVA repeated measures test.

**Table 6:** Comparison of the average knowledge, performance, and total score of oxygen therapy between intervention and control groups in the three-time of the study.

Variables		Group			CI, 95%	
		Intervention group mean ± SD	Control group mean ± SD	*P* value	Lower	Upper
Knowledge (0–30)	Before	20.10 ± 2.59	20.47 ± 2.34	0.512	−1.495	0.752
	After-1 month	24.41 ± 2.06	20.29 ± 2.24	*P* < 0.0001	3.144	5.098
	After-3 months	22.13 ± 2.76	20.24 ± 2.33	0.002	0.729	3.053

Practice (0–24)	Before	18.03 ± 1.67	18.58 ± 1.50	0.132	−1.276	0.170
	After-1 month	21.54 ± 1.46	18.05 ± 1.08	*P* < 0.0001	2.898	4.073
	After-3 months	19.74 ± 1.51	18.63 ± 1.61	0.003	0.400	1.824

Total (0–54)	Before	38.13 ± 3.98	39.05 ± 3.62	0.291	−2.656	0.807
	After-1 month	45.95 ± 2.87	38.34 ± 2.93	*P* < 0.0001	6.288	8.925
	After-3 months	41.87 ± 3.76	38.87 ± 3.30	*P* < 0.0001	1.394	4.613

SD: standard deviation, CI: confidence interval.

## Data Availability

The datasets used and/or analyzed during the current study are available from the corresponding author upon reasonable request
